# Prevalence, causes, and predictors of 30-day readmissions after acute myocardial infarction with ventricular arrhythmias: a cohort study using the 2016–2017 national readmissions database

**DOI:** 10.1097/MS9.0000000000005534

**Published:** 2026-09-08

**Authors:** Adarsh Raja, Muhammad Umer Sohail, Muhammad Ans, Saad Ahmed, Ahsan Feroze, Manaal Ashar, Kehan Ali Rizvi, Muhammad Huzaifa Ahmed Khan, Sara Rahman, Muhammad Umer Hayat, Noor Ul Ain, Laraib Kamran, FNU Pirih, Muhammad Bilal Liaqat, Laksh Kumar, Sumet Kumar, Aayush Chaulagain

**Affiliations:** a Department of Internal Medicine, Shaheed Mohtarma Benazir Bhutto Medical College Lyari, Karachi, Pakistan; b Department of Medicine, Dow University of Health Sciences, Karachi, Pakistan; c Department of Medicine, Fatima Memorial Hospital, College of Medicine and Dentistry, Lahore, Pakistan; d Karachi Institute of Medical Sciences, Karachi, Pakistan; e Jinnah Medical and Dental College, Karachi, Pakistan; f United Medical and Dental College, Karachi, Pakistan; g Karachi Medical and Dental College, Karachi, Pakistan; h King Edward Medical University, Lahore, Pakistan; i Services Hospital, Lahore, Pakistan; j Gujranwala Medical College, Gujranwala, Pakistan; k Rahbar Medical and Dental College, Lahore, Pakistan; l People’s University of Medical and Health Sciences, Shaheed Benazirabad, Pakistan; m Centre for Non-Communicable Diseases (CNCD), Karachi, Pakistan; n Patan Academy of Health Sciences, Lalitpur, Nepal

**Keywords:** 30-day readmission, acute myocardial infarction, healthcare costs, multivariable logistic regression, Nationwide Readmissions Database, predictors of readmission, ventricular arrhythmias

## Abstract

**Background:**

Patients surviving acute myocardial infarction (AMI) complicated by ventricular arrhythmias (VAs) are at high risk of early post-discharge readmissions due to multiple complications. These readmissions worsen clinical outcomes and add substantial financial strain to the U.S. healthcare system. This study investigates the prevalence, causes, and predictors of 30-day readmissions following hospitalization for AMI complicated by VAs.

**Methods and results:**

Hospitalizations for AMI complicated by VAs were identified from the 2016–2017 Nationwide Readmissions Database. Independent predictors of 30-day readmission were analyzed using multivariable survey-weighted logistic regression in the R statistical software (version 4.5.0). Among 93 588 index hospitalizations, 8154 (8.7%) resulted in 30-day readmissions. In-hospital mortality occurred in 16.43% of index admissions and 5.92% of readmissions. Significant predictors of readmission included older age (OR: 1.01; *P* < 0.001), female sex (OR: 1.13; *P* = 0.002), prior heart failure (OR: 1.28; *P* < 0.001), peripheral vascular disease (OR: 1.18; *P* = 0.002), COPD (OR: 1.43; *P* < 0.001), atrial fibrillation (OR: 1.24; *P* < 0.001), diabetes (OR: 1.29; *P* < 0.001), previous MI (OR: 1.12; *P* = 0.009), end-stage renal disease (ESRD) (OR: 1.52; *P* < 0.001), stroke (OR: 1.52; *P* < 0.001), and hospital stay >4 days (OR: 1.94; *P* < 0.001). Higher income quartiles were associated with lower readmission risk. The mean index charges were $151 722, whereas charges per readmission were $64 122.

**Conclusions:**

AMI complicated by VAs is associated with high 30-day readmission rates, driven mainly by noncardiac and select cardiac causes such as heart failure and atrial fibrillation. Enhanced monitoring of high-risk groups may improve outcomes and reduce healthcare costs.

## Introduction

Acute myocardial infarction (AMI), one of the most serious manifestations of coronary artery disease, is associated with increased morbidity, mortality, and impaired quality of life^[^1^]^. Approximately one in six patients who are diagnosed with AMI have an unplanned readmission within 30 days of hospital discharge^[^2^]^. Frequent hospital readmissions result in adverse patient outcomes caused by hospital-acquired infections or complications, and increase the financial burden on both public and private payers^[^3^]^. Under the Hospital Readmissions Reduction Program, the Centers for Medicare and Medicaid Services considers 30-day hospital readmission a measure of patient care quality and penalizes hospitals for high readmission rates^[^4,5^]^. Roughly 18% of all Medicare patients are readmitted within 30 days, amounting to an annual cost of $15 billion. Given the adverse outcomes associated with hospital readmissions, many hospitals nationwide have adopted strategies aimed at minimizing preventable complications during hospitalization. These efforts include improving the quality of patient discharge, ensuring smoother transitions of care after discharge, and enhancing communication and coordination with other healthcare providers to effectively reduce readmission rates^[^6,7^]^. Recent studies suggest that sustained initiatives targeting conditions with high readmission rates, such as AMI and acute heart failure, hold promise in significantly lowering the incidence of unplanned hospital returns. These focused efforts not only improve patient outcomes but also contribute to reducing the financial burden on the healthcare system^[^8^]^.

Given the importance of reducing rehospitalizations, numerous studies have investigated the epidemiology, causes, and predictors of readmission after AMI, identifying several patient-, disease-, and healthcare-related factors involved in early readmission^[^9,10^]^. Although these studies have substantially improved understanding of AMI-specific readmissions, further studies evaluating high-risk subgroups, including patients with AMI complicated by ventricular arrhythmias (VAs), remain insufficient.

VAs, including ventricular tachycardia (VT) and ventricular fibrillation (VF), are highly critical complications of AMI and are associated with significant morbidity and mortality. VT develops in approximately 10% of patients following AMI, and VF remains a major contributor to sudden cardiac death during the acute phase of myocardial infarction^[^11,12^]^. Although multiple studies have extensively investigated the epidemiology, causes, and predictors of readmissions after AMI in the general population^[^3,8,9^]^, data regarding 30-day readmissions among patients with AMI complicated by VAs, which represents a more narrowly defined area of research, remain limited. Therefore, using the Nationwide Readmissions Database (NRD), one of the largest publicly available databases in the United States, we evaluated the prevalence, causes, and predictors of 30-day readmissions following index hospitalizations for AMI complicated by VAs.

## Methods

Due to the public nature of the NRD database, Institutional Review Board (IRB) approval was not required for this study, as no personally identifiable information is included. Furthermore, in accordance with the TITAN Guidelines 2025 for transparent use of AI in scholarly communication, no AI tools were used in the research design, data collection, analysis, or interpretation; AI assistance was limited solely to language refinement during manuscript preparation^[^13^]^.

### Data source

This retrospective cohort study was produced using the NRD, a nationally representative dataset developed by the Healthcare Cost and Utilization Project (HCUP). The NRD is created from the State Inpatient Databases and represents approximately half of all U.S. hospitalizations. The unweighted NRD includes data from around 17 million discharges per year, and, after survey weights are applied, it represents approximately 36 million discharges, excluding rehabilitation and long-term acute care facilities. Discharge weights are used to generate national estimates from the sample data. The NRD contains verified patient linkage numbers that allow tracking of individuals across hospitals within a state; however, the patient linkage numbers do not track the same patient across different years. We applied survey weights, stratification, and clustering information provided by HCUP to generate national estimates that account for the complex sampling design. Informed consent and IRB approval were not necessary because all data were de-identified and publicly available. This study has been reported in accordance with the Strengthening the Reporting of Cohort Studies in Surgery (STROCSS) criteria^[^14^]^.

HIGHLIGHTS

- This is the first nationwide analysis of 30-day readmissions after AMI complicated by ventricular arrhythmias (93 588 hospitalizations).
- In total, 8.7% were readmitted within 30 days – below the approximately 12–14% AMI benchmark–reflecting high early mortality in this cohort.
- Dual burden of disease: In-hospital mortality reached 16.4% at the index admission, compared with 5.9% at readmission.
- The strongest predictors were a length of stay >4 days (OR: 1.94) and ESRD (OR: 1.52); COPD, diabetes, atrial fibrillation, and prior heart failure also increased risk.
- Heavy economic toll: mean charges were $151 722 per index stay and $64 122 per readmission.

### Study population

We included adult patients (aged ≥18 years) with a principal discharge diagnosis of AMI, identified using the International Classification of Diseases, Tenth Revision, Clinical Modification (ICD-10-CM) codes aligned with the AMI pattern (I2101, I2102, I2109, I2111, I2119, I2121, I2129, I213, I214, I219, I21A1, I21A9, I21B, I220, I221, I222, I228, I229). Furthermore, the cohort was restricted to hospitalizations complicated by VAs, defined by the presence of at least one secondary diagnosis code for VT or VF (I470, I472, I4901, I4902). Detailed information on ICD-10-CM codes used for cohort identification is provided in Supplemental Digital Content Table1, available at: https://links.lww.com/MS9/B362. Only non-elective admissions discharged between January and November were eligible to ensure a full 30-day follow-up. We excluded patients who died during the index admission or had missing data on length of stay or discharge timing. Each patient was limited to one index hospitalization per year. Readmissions occurring within 30 days of discharge were identified using the NRD’s synthetic patient linkage variable. Trauma-related hospitalizations were excluded from the readmission pool to minimize unrelated planned returns.

### Patient and hospital characteristics

Baseline patient characteristics included socioeconomic demographics (age, sex, median household income by ZIP code, and primary expected payer), weekend versus weekday admission, and relevant comorbidities. Comorbidities were identified from the Elixhauser Index using corresponding ICD-10-CM codes and included congestive heart failure (CHF), peripheral vascular disease, chronic obstructive pulmonary disease (COPD), type 2 diabetes mellitus (T2DM), hypertension (HTN), end-stage renal disease (ESRD), anemia, smoking, obesity, and alcohol use disorder. Additional clinical risk factors such as atrial fibrillation, previous myocardial infarction, dilated cardiomyopathy, personal history of sudden cardiac arrest, and family history of sudden cardiac death were also identified. Acute in-hospital complications, including acute kidney injury and cardiogenic shock, were identified via ICD-10 codes as well. Discharge status was assessed for non-home discharge (e.g., skilled nursing facility, hospice, other facilities, or death). The length of stay (LOS), reported in days, was modeled as a count outcome. Hospital-related characteristics such as bed size (small, medium, and large), location (urban versus rural), and teaching status (teaching versus nonteaching) were also identified.

### Outcome measure

The primary outcome was the 30-day all-cause readmission rate following discharge from the index hospitalization for AMI complicated by VAs. Readmissions were identified using the NRD’s verified patient linkage numbers, which enable tracking of individuals across hospitals within the same calendar year^[^15^]^. Trauma-related hospitalizations were excluded from the readmission pool to minimize bias from noncardiac, injury-related rehospitalizations. The secondary outcomes included in-hospital mortality, LOS, and total hospitalization charges. In-hospital mortality was defined as death occurring during either the index admission or any readmission. LOS was measured in days for both index and readmission hospitalizations. Total hospitalization charges were obtained from the HCUP TOTCHG variable and converted to costs, then inflation-adjusted to 2017 U.S. dollars using the Consumer Price Index for medical care services.

### Statistical analysis

All analyses were performed in accordance with HCUP guidelines, incorporating survey weights (DISCWT), strata (NRD_STRATUM), and hospital clustering (HOSP_NRD) to account for the NRD’s complex survey design and generate nationally representative estimates. Statistical analyses were conducted using R (version 4.5.0) with the survey and srvyr packages. A two-sided *P*-value < 0.05 was considered statistically significant. Baseline characteristics of index hospitalizations were summarized after weighting and stratified by 30-day readmission status (readmitted vs. not readmitted). Categorical variables were compared using the Rao–Scott adjusted chi-square test, while continuous variables were compared using the design-based Kruskal–Wallis test. Independent predictors of 30-day readmission were assessed using a multivariable survey-weighted logistic regression model. Covariates included demographic characteristics, comorbid conditions, hospital-level factors, and index hospitalization variables. Reference categories were explicitly defined (e.g., male sex, length of stay ≤4 days). Results are presented as odds ratios (ORs) with 95% confidence intervals (CIs).

## Results

### Baseline and descriptive statistics

Of the 93 588 index hospitalizations (73% males vs 27% females) for AMI complicated by VAs, a total of 8154 (8.71%) were readmitted within 30 days. The mean length of stay during index hospitalizations was 6.71 days (95% CI: 6.56–6.85), with a mean charge of $151 722 (95% CI: 146 479–156 966). In contrast, the mean length of stay during readmission hospitalizations was 5.11 days (95% CI: 4.92–5.30), with an associated mean charge of $64 122 (95% CI: 60 824–67 421). Patients who were readmitted were primarily older, with a mean age of 68 years (*P* < 0.001). Among all readmissions, males accounted for a greater proportion than females (70% vs 30%). These patients were also disproportionately represented in the lowest income quartile, with overall, 28% of patients in the 0–25th percentile (*P* < 0.001). The majority of the hospitalizations occurred on weekdays, with more than half (72%) in large metropolitan teaching hospitals. Readmitted patients had a higher burden of comorbidities than those not readmitted, with significantly greater prevalence of heart failure, atrial fibrillation, T2DM, ESRD, acute kidney injury (AKI), anemia, COPD, and previous stroke (all *P* < 0.001). However, cardiogenic shock was more prevalent in index hospitalizations compared to those who were readmitted (23% vs 19%, *P* < 0.001). Patients readmitted within 30 days also had significantly longer initial hospital stays compared to those not readmitted (>4 days: 62% vs 42%, *P* < 0.001). In-hospital mortality was observed in 16.43% of index hospitalizations and in 5.92% of readmissions (Tables 1 and 2).

**Table 1:** Overall baseline characteristics of the study cohort.

Characteristics	Overall^a^		No readmission^a^		Readmission^a^		
	(%)	(*n*)	(%)	(*n*)	(%)	(*n*)	*P*-Value^b^
All patients	100	93 588	91.3	85 433	8.7	8154	–
Patient demographics							
Age, mean (SD), years	65 (13)	–	65 (13)	–	68 (13)	–	<0.001
Female	27.0	24 899	26.0	22 420	30.0	2479	<0.001
Weekend admission	29.0	26 826	29.0	24 525	28.0	2301	0.5
Family history of sudden cardiac death	0.2	157	0.2	140	0.2	17	0.4
Personal history of sudden cardiac arrest	2.1	1967	2.1	1835	1.6	132	0.021
Previous heart failure	16.0	14 637	15.0	12 806	22.0	1831	<0.001
Previous myocardial infarction	16.0	14 660	15.0	13 103	19.0	1557	<0.001
Previous stroke	8.1	7608	7.8	6649	12.0	959	<0.001
Dilated cardiomyopathy	2.1	1933	2.0	1681	3.1	251	<0.001
Current or past smoking history	24.0	22 718	24.0	20 929	22.0	1789	0.001
Atrial fibrillation	26.0	24 384	25.0	21 474	36.0	2910	<0.001
Acute kidney injury	28.0	26 507	28.0	23 825	33.0	2682	<0.001
Cardiogenic shock	23.0	21 514	23.0	19 924	19.0	1590	<0.001
Chronic comorbidities							
Alcohol use disorder	4.5	4255	4.6	3920	4.1	335	0.2
Anemia	24.0	22 820	24.0	20 164	33.0	2655	<0.001
COPD	17.0	15 799	16.0	13 714	26.0	2084	<0.001
Diabetes mellitus	32.0	30 175	31.0	26 755	42.0	3420	<0.001
Hypertension	55.0	51 207	55.0	46 885	53.0	4322	0.022
Obesity	18.0	16 763	18.0	15 252	19.0	1511	0.3
Peripheral vascular disease	9.7	9121	9.3	7951	14.0	1170	<0.001
End stage renal disease	3.8	3518	3.4	2932	7.2	586	<0.001
Median home income							
1st to 25th percentile	28.0	25 656	28.0	23 145	31.0	2511	<0.001
26th to 50th percentile	28.0	25 348	28.0	23 135	28.0	2214	<0.001
51st to 75th percentile	25.0	23 210	25.0	21 242	24.0	1968	<0.001
76th to 100th percentile	19.0	17 905	20.0	16 561	17.0	1344	<0.001
Length of stay category							
≤4 days	56.0	52 626	58.0	49 488	38.0	3137	<0.001
>4 days	44.0	40 962	42.0	35 945	62.0	5017	<0.001

COPD, chronic obstructive pulmonary disease; DM, diabetes mellitus.

^a^ Mean (SD); *n* (%)

^b^ Design-based Kruskal–Wallis test; Pearson`s X^2^: Rao & Scott adjustment

**Table 2:** Weighted baseline characteristics of the study cohort by hospital type and bed size.

Characteristics	Overall		No readmission		Readmission		
	(%)	(*n*)	(%)	(*n*)	(%)	(*n*)	*P*-Value
Hospital location							
Metropolitan, non-teaching	20.5	19 185	20.8	17 769	19.2	1566	0.8
Metropolitan, teaching	10.3	9639	10.3	8799	10.2	831	0.8
Non-metropolitan	9.1	8516	9.4	8035	7.8	636	0.8
Hospital bed size							
Small bed size	12	11 230	12	10 252	12	978	0.7
Medium bed size	28	26 205	28	23 921	27	2201	0.7
Large bed size	61	57 088	61	52 114	61	4974	0.7

### Multivariate analysis (Predictors)

The multivariate regression analysis revealed several statistically significant predictors of 30-day readmissions (all with *P* < 0.05), including increasing age [odds ratio (OR) 1.01; 95% confidence interval (CI), 1.01–1.01; *P* < 0.001], female sex (OR: 1.13; 95% CI: 1.04–1.22; *P* = 0.002), prior CHF (OR: 1.28; 95% CI: 1.17–1.40; *P* < 0.001), peripheral vascular disease (OR: 1.18; 95% CI: 1.06–1.31; *P* = 0.002), COPD (OR: 1.43; 95% CI: 1.31–1.57; *P* < 0.001), atrial fibrillation (OR: 1.24; 95% CI: 1.14–1.35; *P* < 0.001), T2DM (OR: 1.29; 95% CI, 1.20–1.40; *P* < 0.001), prior MI (OR: 1.12; 95% CI: 1.03–1.23; *P* = 0.009), ESRD (OR: 1.52; 95% CI: 1.29–1.78; *P* < 0.001), and prior stroke (OR: 1.23; 95% CI: 1.09–1.38; *P* < 0.001). Additionally, LOS of more than 4 days at index admission was also associated with 30-day readmission (OR: 1.94; 95% CI: 1.78–2.11; *P* < 0.001). However, prior history of cardiogenic shock (OR: 0.67; 95% CI: 0.61–0.73; *P* < 0.001) and sudden cardiac arrest (OR: 0.72; 95% CI: 0.56–0.92; *P* = 0.009) were associated with lower 30-day readmissions. Belonging to the medium (51st–75th percentile; OR: 0.90; 95% CI: 0.81–1.00; *P* = 0.041) and high median income quartiles (76th–100th percentile; OR: 0.79; 95% CI: 0.71–0.88; *P* < 0.001) was also associated with lower readmissions (Table 3).

**Table 3:** Predictors of 30-day readmission following AMI and ventricular arrhythmias.

Variable	Odds ratio	95% CI	*P*-Value
Patient demographics			
Age, per 1-year increase	1.01	(1.01, 1.01)	<0.001
Female sex	1.13	(1.04, 1.22)	0.002
Weekend admission	1.02	(0.95, 1.10)	0.6
Median home income (reference: 1st to 25th percentile)			
26th to 50th percentile	0.91	(0.83, 1.01)	0.066
51st to 75th percentile	0.90	(0.81, 1.00)	0.041
75th to 100th percentile	0.79	(0.71, 0.88)	<0.001
Hospital characteristics:			
Hospital bed size (reference: small)			
Medium	0.93	(0.81, 1.06)	0.2
Large	0.93	(0.82, 1.04)	0.2
Teaching status (reference: metropolitan, non-teaching)			
Metropolitan, teaching	0.93	(0.86, 1.01)	0.068
Non-metropolitan	0.96	(0.78, 1.18)	0.7
Comorbidities			
Alcohol abuse	0.90	(0.75, 1.07)	0.2
Current or past smoking history	1.00	(0.90, 1.10)	>0.9
Anemia	1.04	(0.96, 1.13)	0.3
COPD	1.43	(1.31, 1.57)	<0.001
DM	1.29	(1.20, 1.40)	<0.001
Hypertension	1.02	(0.95, 1.10)	0.5
Obesity	0.96	(0.88, 1.05)	0.4
PVD	1.18	(1.06, 1.31)	0.002
Previous heart failure	1.28	(1.17, 1.40)	<0.001
Previous myocardial infarction	1.12	(1.03, 1.23)	0.009
Previous stroke	1.23	(1.09, 1.38)	<0.001
Family history of sudden cardiac death	1.54	(0.71, 3.35)	0.3
Personal history of sudden cardiac arrest	0.72	(0.56, 0.92)	0.009
Dilated cardiomyopathy	1.11	(0.90, 1.37)	0.3
Atrial fibrillation	1.24	(1.14, 1.35)	<0.001
End stage renal disease	1.52	(1.29, 1.78)	<0.001
In-hospital complications			
Acute kidney injury	0.99	(0.91, 1.07)	0.8
Cardiogenic shock	0.67	(0.61, 0.73)	<0.001
Length of stay category (reference: ≤ 4)			
>4	1.94	(1.78, 2.11)	<0.001

CI, confidence interval; COPD, chronic obstructive pulmonary disease; DM, diabetes mellitus; PVD, peripheral vascular disease.

## Discussion

In this observational analysis of a large, national cohort of hospitalizations for AMI complicated by VAs, we identified a 30-day readmission rate of 8.7% among index hospitalizations. Mortality during the initial hospitalization was markedly high at 16.43%, compared with 5.9% during readmissions. Furthermore, the index hospitalizations were more resource-intensive and longer, with a mean length of stay of nearly a week and mean charges exceeding $150 000. In contrast, readmissions had a shorter median length of stay of about 5 days and substantially lower charges of $64 122. Taken together, these findings highlight the twofold burden of early mortality and recurrent hospitalizations in such a high-risk population and illustrate the clinical and economic burden of AMI complicated by VAs at a national level.

To the best of our knowledge, this is among the first nationwide studies to describe 30-day readmissions in AMI complicated by VAs using the NRD. Notably, the readmission rate for our cohort was significantly lower than the 14% benchmark reported in the literature for Medicare patients hospitalized for AMI^[^16^]^. Similarly, in a systematic review and meta-analysis of 14 studies (including registry and cohort studies) involving AMI patients from multiple countries, the pooled results revealed a 12% 30-day readmission rate (with rates ranging from 10% to 15% in the included studies)^[^17^]^. This contradiction can be related to the extremely high early mortality associated with VAs, which reduces the final pool of patients who survive to be at risk of rehospitalization. The sizable variation in mortality between index and readmission hospitalizations is consistent with other national data, which have indicated that concentrated mortality occurs during the index hospitalization, while subsequent readmissions, although common, are less likely to have a fatal outcome^[^18^]^. Medicare analyses have also shown that the median time to rehospitalization after AMI is approximately 10 days post-discharge^[^19^]^. Collectively, this indicates a higher risk of future hospitalization for patients who survive to be discharged from a complicated AMI hospitalization, particularly in the first few weeks, despite the immediate mortality risk being lower. This reinforces the critical need for targeted transitional care planning and close post-discharge monitoring to mitigate early readmission risk.

The longer and more expensive index stay can be attributed to the intensive monitoring, procedures, and interventions often required in this setting^[^20^]^. Even though readmissions are shorter and less expensive, they remain noteworthy contributors to overall healthcare spending. Previous work has suggested that post-AMI readmissions cost Medicare more than a billion dollars annually^[^17^]^. Our findings further cement this claim, showing that even within a subset of AMI complicated by VAs there is an extraordinary economic toll associated with repeat hospitalization. Since 30-day readmission is a publicly reported performance measure for the Centers for Medicare & Medicaid Services Hospital Readmissions Reduction Program, our findings are directly pertinent to health systems, payers, and policymakers^[^21,22^]^. In this high-acuity patient population, interventions to improve discharge planning, enhance early patient follow-up, and target resources to high-risk survivors may prove to be beneficial in reducing readmissions and downstream costs.

Through multivariable regression analysis, we identified certain key predictors associated with a higher risk of 30-day readmission, such as LOS >4 days, ESRD, COPD, T2DM, previous HF, atrial fibrillation, previous stroke, peripheral vascular disease, female sex, previous MI, and increasing age. Our results mirror findings from a previous analysis of the NRD evaluating readmissions after catheter ablation of MI-linked VT (from 2010 to 2015)^[^23^]^. Cheung *et al* identified increased LOS, pulmonary hypertension, and chronic pulmonary disease, among other factors, as significant predictors linked with an increased risk of readmission. Another NRD-based study found diabetes, chronic lung disease, renal failure, and cancer as key factors associated with rehospitalization only after AMI^[^24^]^.

Among the predictors, a prolonged index LOS stood out as an important factor for rehospitalization according to our results. Although a longer LOS itself does not lead to a higher risk of readmission, it correlates with greater clinical complexity, severity of condition, and increased hospital-acquired complications. These factors can eventually lead to early readmission, since patients with prolonged LOS are usually more vulnerable^[^25–27^]^. Furthermore, our study reinforces ESRD as a potent risk factor for readmissions among AMI patients complicated by VAs. According to an NRD analysis, ESRD is associated with a twofold increased risk of rehospitalization among AMI patients^[^28^]^. Existing literature states that ESRD elevates the risk of VAs due to autonomic dysfunction, left ventricular hypertrophy, endothelial impairment, and ischemic liability, thereby predisposing patients to early decompensation^[^29,30^]^. Additionally, ESRD is correlated with poor short-term outcomes, including prolonged hospital stays^[^31^]^. Altogether, these factors increase the likelihood of readmissions among patients with AMI and VAs who also have a history of ESRD. Another patient-related characteristic connected with increased rehospitalization risk is COPD as a comorbidity. According to a study, patients with COPD coupled with arrhythmias had a threefold higher risk of readmission^[^32^]^. Mechanisms largely associated with this include increased systemic inflammation, sympathetic stimulation, and hypoxia, which occur due to worsening COPD, thereby triggering arrhythmias^[^33–35^]^. In addition, diabetes was also linked with higher readmission rates. This finding is corroborated by a study highlighting diabetes as a significant, multifaceted risk factor in AMI-related readmissions^[^17^]^. Vascular damage, coupled with an elevated VT risk resulting from QT-interval prolongation, and attenuated healing and recovery result in reduced post-discharge resilience among patients with a history of diabetes, eventually leading to early readmission^[^17,36,37^]^. These findings highlight the importance of timely post-discharge contact and clinic follow-up, alongside strict guideline-directed discharge stability benchmarks, especially among high-risk individuals^[^38^]^. Furthermore, a multidisciplinary team involving nephrologists, pulmonologists, endocrinologists, and hospitalists should be utilized in managing AMI patients complicated by VAs. These interventions might help reduce 30-day rehospitalizations.

When discussing gender, female sex was associated with a higher readmission risk, and this finding is consistent with results from prior NRD-based studies^[^39^]^. Reasons associated with this disparity include a higher burden of comorbid conditions among women, a reduced likelihood of receiving lifesaving interventions like Percutaneous Coronary Intervention (PCI) and greater psychosocial vulnerability^[^40–42^]^. Increasing age was also significantly associated with readmission risk; this finding is consistent with prior studies, which report that those aged 75 years or older had higher odds of readmission; however, according to the study involving AMI patients undergoing PCI, those aged 65–75 years had a lower risk of all-cause readmission^[^43^]^. Among cardiac causes, previous heart failure was one of the significant predictors of higher 30-day readmission risk; this is evidenced by prior studies as well; a meta-analysis reported HF as an important comorbidity that increased the risk of rehospitalization^[^17^]^. The history of heart failure is tied to reduced ejection fraction, which further increases the susceptibility to decompensation, hence positioning it as a key predictor of an elevated readmission risk^[^44^]^. Other potent factors included atrial fibrillation, previous stroke, peripheral vascular disease, and previous MI. These conditions induce hemodynamic instability and exacerbate the already compromised patients’ health, which manifests as early readmission. Atrial fibrillation adds to the impaired cardiac rhythm by causing recurrent arrhythmias and eventually stroke, hence adding to the post-discharge clinical instability, resulting in readmission^[^45,46^]^. A comprehensive strategy that involves prompt revascularization using PCI and coronary artery bypass grafting (CABG) alongside timely initiation of guideline-directed medical therapy after discharge [comprising beta-blockers, angiotensin-converting enzyme (ACE) inhibitors, statins, mineralocorticoid receptor antagonists (MRAs), etc.], coupled with early post-discharge cardiology follow-up, might help in mitigating the readmission burden among high-risk individuals with index hospitalizations due to AMI complicated by VAs^[^47–51^]^.

We also identified that belonging to the middle (26th–50th percentile) and high (76th–100th percentile) median-income quartiles was associated with lower readmission rates. A similar trend has been observed in prior studies focusing on AMI-related readmissions^[^52^]^. Since higher-income individuals have better post-discharge access to medications and follow-up, this reduces the readmission risk^[^53^]^. Although lower-income individuals had reduced readmission risk according to our results, the finding was non-significant. This is largely attributed to the fact that lower-income patients have greater in-hospital or post-discharge mortality (competing risk bias), which reduces the number of patients who are susceptible to readmission, hence leading to underestimation of the actual burden of readmissions in this cohort^[^54^]^. Similarly, cardiogenic shock and cardiac arrest were linked to lower readmission rates. However, these results should be interpreted cautiously, since patients who died during the index hospitalization were excluded, and, hence, survivor bias may have impacted these associations. Patients exhibiting more severe clinical manifestations might have experienced greater in-hospital mortality, and, as a result, were not included in the readmission cohort. Consequently, the observed inverse association does not imply a protective effect of cardiogenic shock or sudden cardiac arrest on readmission risk and may reflect the selection of a healthier subgroup among those surviving to discharge. Additionally, competing risk bias should also be considered when interpreting this inverse association. Patients with serious complications, including cardiogenic shock or sudden cardiac arrest, belong to a high-risk subgroup that might experience greater post-discharge mortality, thereby establishing death as a competing risk for readmission. As a result, lower observed readmission rates in these groups may not reflect a lower burden of illness or a genuinely reduced risk of readmission but rather the fact that some patients experience mortality before a readmission event can occur. Future research should employ competing risk models to avoid underestimation of readmission burden.

### Clinical implications and potential strategies to reduce readmissions

The risk factors observed in our analysis might help clinicians screen patients who are at a greater risk of early readmission following AMI complicated by VAs, so that targeted interventions can be implemented.

First, executing a transitional-care program might greatly reduce the burden of readmissions among high-risk patients, including those with prolonged hospitalization (LOS >4 days), prior heart failure, atrial fibrillation, prior myocardial infarction, prior stroke, peripheral vascular disease, and advanced age. A recent study has shown that an early patient follow-up after 7–30 days of discharge is linked to markedly lower rehospitalization rates among patients with heart failure and AMI^[^55^]^. Therefore, it is imperative that early cardiology review, medication reconciliation, and reinforcement of discharge instructions are emphasized for these high-risk patients. Additionally, a multidisciplinary approach within the transitional-care program, consisting of home visits, nurse-led follow-ups, and organized outpatient management, has been shown to reduce readmissions among high-risk cardiovascular patients^[^56^]^.

Second, patients with T2DM and ESRD need closer post-discharge monitoring due to the complex nature of their medical needs and a greater risk of cardiovascular complications. Among such patients, early evaluation of glycemic control, compliance with medication, volume status, and dialysis adequacy may help avoid potential readmissions. Third, an integrated cardiopulmonary follow-up strategy may reduce the readmission burden among patients with COPD. Potential actions include optimization of inhaler therapy before discharge, smoking cessation interventions, vaccinations, and referral to pulmonary rehabilitation ^[^57^]^.

Fourth, older individuals and female patients may require further discharge support due to a higher comorbidity burden, frailty, and barriers to healthcare access. Hence, early outpatient follow-up might mitigate the readmission risk among these individuals. Lastly, eligible patients should be strongly encouraged to participate in cardiac rehabilitation, as it is associated with a better prognosis and lower readmission rates in cardiovascular patients through optimization of secondary prevention measures, exercise training, risk-factor modification, and improved adherence to guideline-directed medical therapy^[^58^]^.

### Study strengths and limitations

When discussing the strengths of our analysis, it should be noted that this study includes a substantial number of hospitalizations, which are drawn from real-world data. However, certain key limitations should be considered while interpreting our findings. Because this is an observational, nonrandomized analysis, the risk of unmeasured confounding cannot be fully eliminated, even though we adjusted for several confounding variables. Furthermore, since the NRD lacks data regarding some important clinical and procedural factors, including the site of vascular access (femoral vs. radial), post-interventional medications such as anticoagulants and antiplatelets, and stent type (e.g., bare-metal vs. drug-eluting), this limits the possibility of adjusting for treatment-specific variations that may lead to differences in post-discharge outcomes. Alongside this, information related to complete revascularization, which strongly impacts outcomes in AMI, was not available^[^59,60^]^. Additionally, as readmissions cannot be tracked across different states or across calendar years, this leads to underestimation of the actual numbers. Also, ICD-10-CM coding errors and bias (attrition bias, selection bias, etc.) are known limitations of administrative databases like the NRD. Lastly, since the sample size included large numbers, coupled with the large number of factors that were assessed in the adjustment model, the quantitative significance of certain predictors with confidence intervals nearing 1 might have resulted by chance (i.e., type I error). An approach that balances the statistical significance of such variables with effect size, the quality of the research, and results from other studies should help reduce such errors.

## Conclusion

AMI complicated by VAs is associated with high rates of readmissions within 30 days, mainly attributable to predictors like ESRD and COPD. The risk of readmission is also associated with certain baseline patient and hospital characteristics. Further research is warranted to develop enhanced, clinically integrated predictive models to enable more accurate identification of high-risk patients and reduce avoidable rehospitalizations.

## Data Availability

The dataset supporting the conclusions of this article are included in this article.
